# Spectral technique for detection of changes in eggshells caused by *Mycoplasma synoviae*

**DOI:** 10.3382/ps/pez150

**Published:** 2019-04-19

**Authors:** Zofia Lorenc, Sławomir Paśko, Olimpia Kursa, Anna Pakuła, Leszek Sałbut

**Affiliations:** 1 Institute of Micromechanics and Photonics, Faculty of Mechatronics, Warsaw University of Technology, 02-525 Warsaw, Poland; 2 Department of Poultry Diseases, National Veterinary Research Institute, 24-100 Puławy, Poland

**Keywords:** spectral technique, *Mycoplasma synoviae*, transmittance spectrum, classification tree method CTM, binary classification

## Abstract

*Mycoplasma synoviae* (MS) is a major pathogen in chicken and turkeys, causing subclinical infection. MS infections are highly prevalent and may potentate and be involved in sinovitis, respiratory syndromes, as well as lead to eggshell apex abnormality (EAA). A deformed, inhomogeneous eggshell is susceptible to cracks and breaks through which microbes get in and additionally entails higher water loss in the egg during the entire incubation process.

Not all eggs with eggshell apex abnormality possess characteristic deformation and that is why some eggs may be incorrectly classified during a visual inspection. To minimize the above risk, the spectral VIS technique and the analysis based on the classification tree method—CTM is proposed.

The method makes use of specially defined parameters extracted from the shape of transmittance spectra of eggshells. Directional coefficients of the lines adjusted to the specific ranges of the transmittance spectrum are used in the process of classifying samples as those from MS-carrying hens and from healthy hens. Three CTM-based classifiers were created for a group of white, brown, and mixed shells. After comparing, it can be concluded that the best results were obtained for the group of brown shells (accuracy 88%, specificity 88%, and false negative rate 13%).

The authors present a non-invasive spectral method that utilizes eggshells, i.e., the natural waste from chicken farms. The method enables entering data into the classifiers described in the article. The process provides an opportunity to correctly assign, the examined shell to the group of shells with increased risk—with approx. 86% accuracy. This means that, if a few of such results are registered, the herd is eligible more specific studies targeting MS bacteria. Regular spectral testing can support the detection of egg lesions in MS positive flocks.

## INTRODUCTION

Evaluation and categorization of eggs is widely used in the food industry. For this purpose various methods are used with light-based ones playing an important role among them. The US Department of Agriculture was the precursor of the studies using spectroscopy in egg quality assessment since 1949 (Norris, 1992). The assessment of eggs by humans is a bottleneck on the production line, therefore, for the classification of eggs more and more often automated systems are used such as OptiGrader from Sanovo, which is able to analyze up to 216,000 eggs within an hour (Sanovo Technology Group, 2018). Apart from being inefficient the assessment by an observer is also subjective and unstable. Wei and Bitgood (1990) were one of the first that took objective evaluation of eggs under consideration in research using two colorimeters for this purpose. Because the color of the eggshell is important from an economic point of view, various types of egg-type chickens were tested to determine the variance of colors in each group.

Testing the color of the egg and its pigmentation is used not only for the classification within the production but it can also be used for other purposes. It has been shown on the example of a brown flock that there is a relationship between pigmentation and the state of health of the individuals in the flock, as well as stress it was exposed to (Mertens et al., 2009, 2010). The authors used visible–near infrared transmission spectroscopy to reach this aim. The study of pigmentation of the bird's egg shell was also carried out using Raman spectroscopy (Thomas et al., 2015).

Determining a relationship between certain parameters and output data is in many cases difficult. In such cases, the data mining algorithms developed for over 50 yr are often used (Loh, 2014). Such method was applied by Çelik et al. (2016), who were looking for Japanese quail egg traits that combine with high fertility rate. Using the CART (classification and regression tree) algorithm, they showed that the highest fertility ratio—90.9% is achieved for eggs of very specific quails. Before, the method of classification tree (CTM) was used by Karabag et al. (2010) for determination of factors affecting the hatching from eggs of Chukar Partridge. They took for analysis the weight, volume, length, and width of the eggs and the accuracy of the analysis associated with the use of CTM for these parameters was 75.6%. CTM as a binary classifier is structurally very simple and easy to visualize what is very important when interpreting the results.

The list of factors finally classifying the egg as defective was presented by the Chukwuka et al. (2011) together with many other useful information associated with the evaluation of eggs. An example of a solution detecting egg shell defects was the expert system proposed by the Omid et al. (2013), where machine vision and fuzzy inference system were integrated. Skillfully creating rules for the fuzzy part, they achieved a general correct classification rate for defect classification of 95.4%. The analysis of egg shell defects is of key importance in the poultry industry due to the protective function it performs (Solomon, 2010). In addition to defects, the freshness of eggs is also assessed. For this purpose visible–infrared transmittance spectroscopy can be used. The analysis of the obtained spectrum can be carried out in many ways. For example, Lin and coworkers in order to create a model, at first processed the data using the principle (Mehdizadeh et al., 2014) component analysis and independent component analysis, and then used the algorithm combining artificial neural network with genetic algorithms (Lin et al., 2011). The accuracy of designed system was 95.4%. Better results were achieved by combining principal components analysis, genetic algorithm and back propagation multilayer perceptron with artificial neural network with one hidden layer (Mehdizadeh et al., 2014). Researchers achieved 100, 81.2, and 100% accuracy for high, medium, and low quality eggs. The freshness of eggs can also be examined using hyperspectral imaging (Suktanarak and Teerachaichayut, 2017). The authors analyzed the reflection of light in the range of 900 to 1,700 nm from the egg shell, trying to connect it with the help of the partial least squares regression with the Haugh unit, an index commonly used to determine egg freshness. Due to this solution, they were able to determine Haugh unit at each point of the tested object.

Eggshell quality has a major economic impact on commercial egg production—broken and cracked eggs mean economic loss. The eggshell protects the embryo from mechanical damage and regulates gas exchange between the developing embryo and the external environment, it also prevents contamination by bacteria and other pathogens. Finally, the eggshell provides a source of nutrients, primarily calcium, to the developing embryo (Yoho et al., 2008). The vital nature of shell quality for the production of eggs justifies why it is so important to understand the factors that can affect it. Pathogenenicity of some *Mycoplasma synoviae* strains can cause considerable economic loss in the poultry industry as this microorganism is known for reducing egg production (Mohammed et al., 1987; Catania et al., 2010). In recent years, the occurrence of strains of MS that induce eggshell apex abnormality (**EAA**) and egg production losses was reported in the Netherlands (Feberwee et al., 2009), Italy (Catania et al., 2016), Germany (Ranck et al., 2010), and Brazil (Brandão et al., 2014). The EAA is characterized by an altered shell surface, shell thinning, increased translucency, cracks, and breaks in the eggshell—and such quality and ultrastructure wise changes were also observed in Polish flocks (Kursa et al., 2019). The natural or experimental MS infection in chickens causing EAA has been confirmed in different reports (Mohammed et al., 1987; Feberwee et al., 2009; Catania et al., 2010; Gole et al., 2012; Kursa et al., 2019). When influencing the structure of the egg shell, the MS bacterium modifies its local thickness, making it more susceptible to cracks. The thickness of the egg shell can be measured with the use of Optical Coherent Tomography, among others, which determines the exact distribution of the shell thickness for the entire surface. Data on the thickness and its variability allow to look more closely at the problems analyzed so far in many experiments in this area (Sabuncu and Akdogan, 2014). Unfortunately, measurement with this method is time-consuming and the measured area is very small, what makes it unsuitable for use in production.

In this article, the VIS spectral measurements are used along with the binary classifiers assigning chicken egg shells to the group of MS infected or healthy.

## MATERIALS AND METHODS

### Samples

During the research 60 brown chicken eggshells and 60 white shells were tested. In each group, half of the eggs came from hens infected with MS and half from healthy hens. All samples were sent by National Veterinary Research Institute in Pulawy. Infected birds had symptoms associated with mycoplasmal infections and produced eggs with anomalies had the demarcation zone at the apex of the egg, the shell thinning and increased translucencyin different areas of the eggshell. The changes were visible with the naked eye and during the candling. Normal eggs come from control groups. In order to prepare the sample for the experiment, the specific procedure was carried out consisting of breaking of an egg, rinsing the shell under a stream of tepid, running water and leaving it to dry for 3 D.

**Figure 1.**
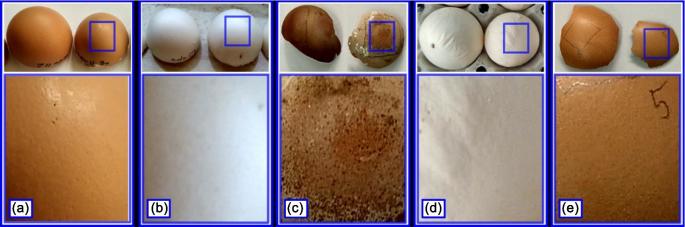
Examples of analyzed chicken eggshells samples: (a) brown eggshells from healthy hens; (b) white eggshells from healthy hens; (c) brown eggshells from hens infected with MS; (d) white eggshells from hens infected with MS; (e) with MS but without visible deformations (visible in candling).

Figure 1 presents selected cases of MS that clearly show what effect this bacteria has on the external appearance of an eggshell. Only some of the eggs laid by infected chickens have such visible changes in shape and color (Figure 1c and 1d). Eggs with changes visible only during the candling are shown in Figure 1e.

### Optical VIS Spectral System and Parameters Generation

**Figure 2.**
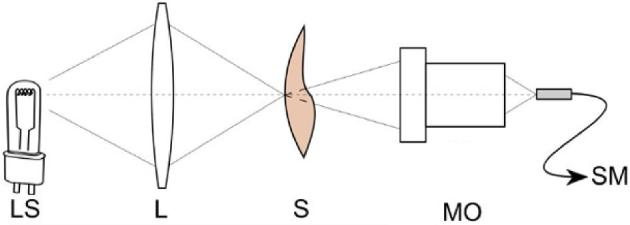
Scheme of a custom-made optical system for transmittance measurement. LS—light source, L—lens, S—sample (eggshell), MO—microscope objective, SM—pigtailed spectrometer.

Prepared samples were placed in a custom-made optical system for transmission measurement (Figure 2). The spectrum of light transmitted through the shell was recorded. In the presented system the light source (**LS**) is an incandescent lamp with continuous spectrum of 400 nm (the used Thorlabs CCS100 spectrometer operates from 350 nm) to the end of the spectrometer range (750 nm). The maximum value of intensity is for wavelength of 640 nm. The source has a sufficiently high luminance (∼3×10^6^ cd/m^2^), which allows measurements in the described system. The beam is focused on the eggshell (S) into an about 1 mm diameter spot by the lens (L) with focal length of 35 mm. Microscope objective focused transmitted beam on optical fiber facet connected to compact spectrometer Thorlabs CCS100 (SM).

The measured areas such as the pointed tip, the oval tip, and the specified part of a side of an egg have been distinguished in a shell of a single egg. In case of a different geometrical structure of a shell, e.g., visible corrugations the changed fragment was considered an additional measurement surface. Five spectra registrations have been carried out in each of the measurement areas. The integration time used during the measurements ranged from 300 to 1,000 ms, depending on the color of a shell. A total of 2,541 spectral measurements of shells have been made.

To obtain the sample transmittance (T_S_) the recorded sample intensity (I_S_) was divided by the source intensity (I_LS_) (T_S_ = I_S_/I_LS_). The sample transmittance is independent of the LS spectrum that illuminates the sample, however, in our case, this procedure does not allow comparison of transmittances calculated from subsequent measurements. The introduction of unwanted diversification of recorded intensities during the measurement series could be caused by the following: i) differentiation of samples in terms of thickness, ii) shape of samples preventing the placement of subsequent shells in perfectly repetitive setup in the system, and iii) imperfection of the LS and its possible fluctuations in intensity over time, mainly among individual test groups (there were several-days differences between the measurement cycles of individual groups of shells). To reduce this effect the results were normalized in the range of 0 to 1. Thanks to such procedure, it is possible to compare shapes of transmittance curves without a thorough analysis of the intensity values in the recorded waveform. This justifies the creation of parameters that classify charts not based on information about intensity values. The authors decided to determine the spectral discrimination parameters that only use information on the geometrical characteristics of registered transmittances. In this way, they made the features independent of the LS instability, the non-ideal placement of a sample and the different thickness of shells during all measurements.

**Figure 3.**
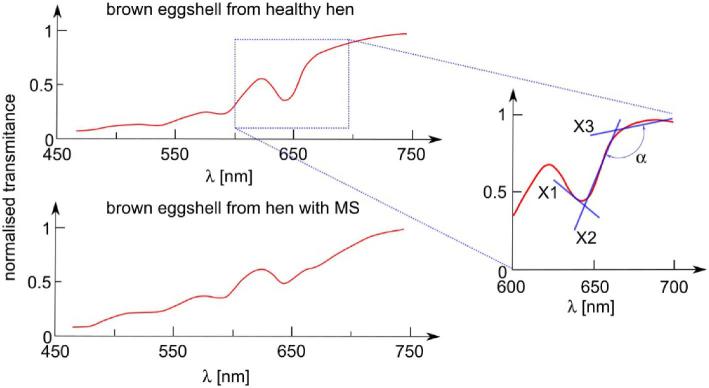
The graphical interpretation of parameters X1, X2, X3, and on exemplary transmittance charts of brown eggshells from MS-infected and healthy hen.

After visual analysis of the calculated transmitting spectra, the authors generated parameters (X1, X2, X3, and α) described by directional coefficients of the straight lines adjusted to the specific narrow ranges (645 to 660 nm, 660 to 675 nm, and 675 to 690 nm, respectively) of the shape of transmittance spectrum and angle between lines X2 and X3. The graphical interpretation of the described parameters is shown in Figure 3.

The creation of a binary classifier that uses the designated parameters as input data enabled the process of effective classification of shells to those from the chickens infected with MS and those from the healthy chickens.

## RESULTS AND DISCUSSION

### Transmittance

**Figure 4.**
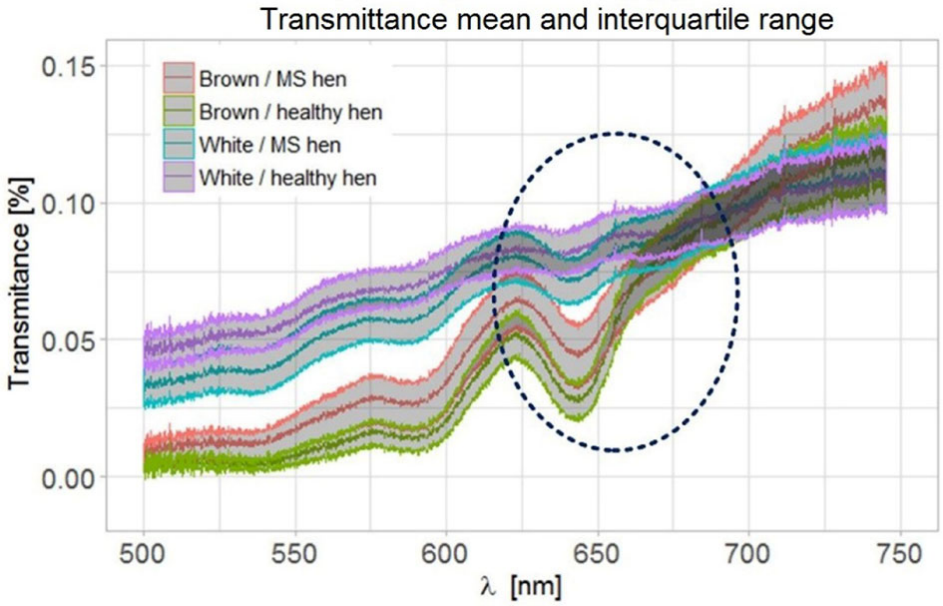
The darkest lines symbolize average transmittance in given test groups (eggshell color and chicken health status were taken into account), the interquartile range (IQR) of each group is marked by gray area. The dashed line marks the area of the largest variation (in geometrical terms) of the graphs among one another.

A significant part of the beam that reaches the sample is reflected and absorbed, approximately 0.1% of the light goes through the shell. Fortunately, this is enough to find significant differences in transmission spectra of eggshells from healthy and MS infected hens. In order to visualize these differences a graph of transmittances of all the examined shells has been created. Eggshell color and chicken health status were taken into account. Figure 4 shows the average transmittance in a given test group and the interquartile range of the registered transmittances. The transmittance level below 0.15% is a result analogous to that reported by Shafey et al. (2004).

The presented statistical dispersion of measurements allows a visual assessment of shell classification. There is a noticeable variation in the level of intensity among classes (this issue is described in more detail in the chapter “Optical VIS spectral system and parameters generation”), which was deliberately not included. The authors limited the generation of features to the geometric aspects of transmittance graphs. In Figure 4, the dashed line marks the area of the largest variation (in geometrical terms) of the graphs towards one another. The features described above have been set in the marked range.

### Features Classification and Evaluation

**Figure 5.**
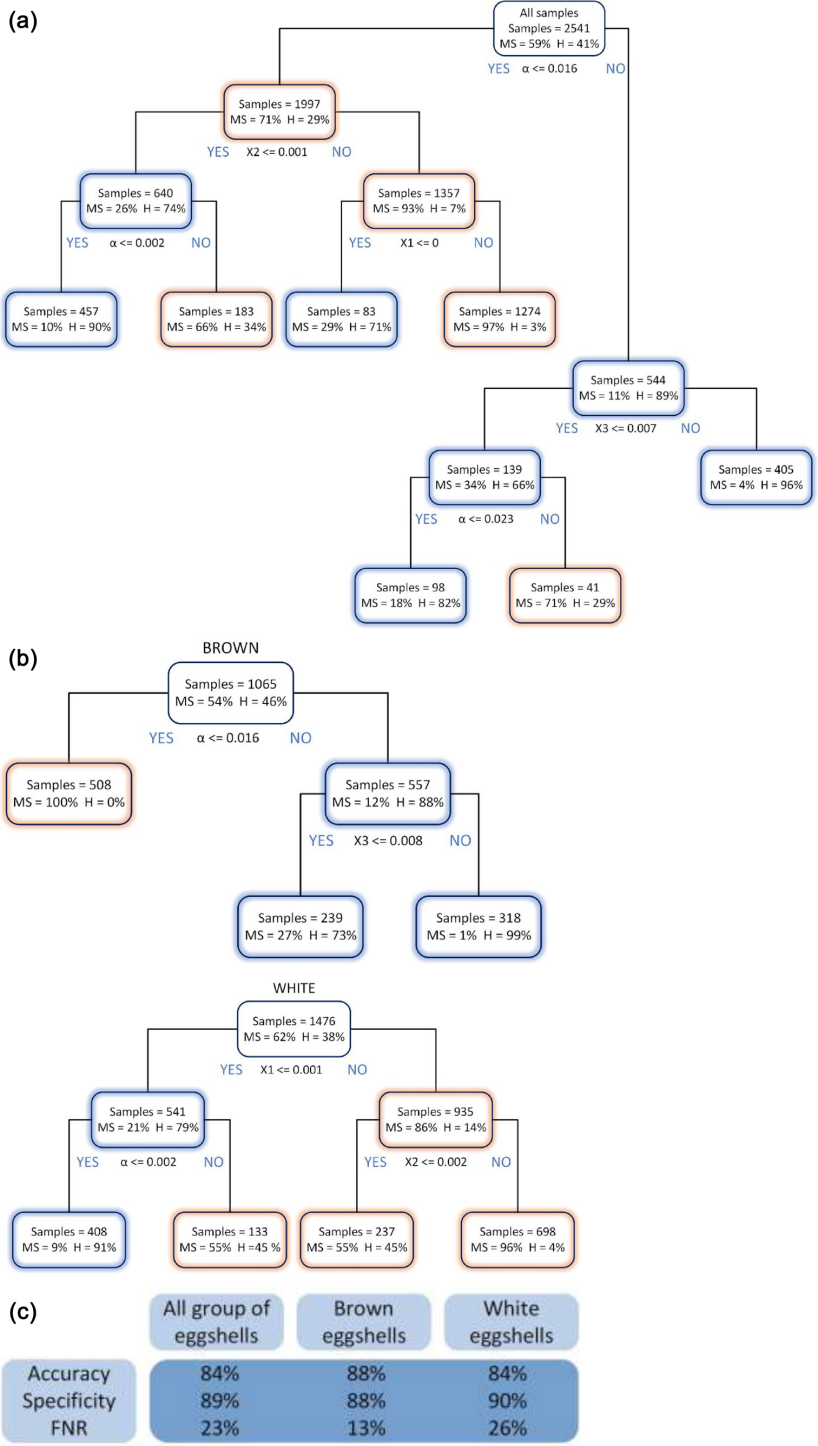
(a) Classification tree of white and brown shells; (b) Classification trees of just brown samples and a group of just white samples; (c) Classification metrics calculated from confusion matrices for three discussed cases.

Three cases of CTM were analyzed—mixed samples of white and brown shells, a group of only brown samples and a group of only white samples (Figure 5b). From a practical point of view, in many cases one breed of hens lays eggs of a specific color, so grouping them in terms of this characteristic is often not problematic. Three selected classification metrics calculated from confusion matrices for all the cases discussed are shown in Figure 5c.

The decision trees are constructed by splitting the whole data into nodes based on yes/no answers of a classification rule values of the predictors shown below the rectangle—node. In each node information is given on the number of samples and the percentage of eggshells from the hens with MS (marked MS) and eggshells from the healthy hens (marked H). The sum of samples in one row is the number of all samples tested. The nodes with the predominant number of samples assigned as MS are marked in red, those with the predominant number of H-assigned samples—in blue. All decisions trees were generated using the Gini method nonlinear combinations. A 5-fold cross-validation analysis was performed as an initial evaluation of the test error of the algorithm. Briefly, this process involves splitting up the dataset into 5 random segments and using 4 of them for training and the fifth as a test set for the algorithm. The key parameters in the presented decision trees are the parameters and X1. Those are the ones that occur in the root node.

Analyzing only CTM graphs, it can be seen that the best results, using proposed method, are achieved for a group of brown eggs. Only one division criterion is needed (α ≤ 0.016) for the root node so that the model shows that 100% of such samples are correctly classified as eggshells from the hens with MS. In the proposed model, in other cases (α > 0.016) of the actually infected samples, only 13% were incorrectly classified as Healthy—**FNR** (false negative rate). In the group of mix-colored shells the value is 23%, while in the white shell group 26%.

In the case of the decision tree describing the group of white shells, there is no parameter that allows samples to be classified as properly as in the brown group. However, the specificity parameter—describing the percentage of correctly qualified actually healthy samples as predicted healthy in the group of actually healthy has a value of 90% for white shells, which is the best result from the analyzed cases (for mixed shells it is 89%, for the group of brown shells 88%).

For a group in which about half of the samples are considered positive (eggs from infected chickens) the accuracy of 84% was achieved for mixed shells—brown and white. It was 88% for brown shells, and 84% for white shells. Hence, in the case studied, CTM made obtaining the best results in the group of brown shells possible. The presented results of assignments along with classification metrics do not fully meet the expectations of the authors, because the data entered into the algorithm may be burdened with an error impossible to be eliminated in conditions of real breeding. It cannot be said that all the eggs in the group labeled as infected came directly from the hens with MS. It is enough that 10% of the blood samples of the examined 60 pieces of hens show a positive result for the entire herd to be considered infected. However, the herd sensitivity is dependent on seroprevalence at a flock level and on the number of samples taken to the serological methods. Subclinical infections of MS can spread quickly after introduction to a farm. For this reason, an egg derived from an MS-infected farm could be laid by a hen that is still healthy or in which the bacterium did not make such changes to be visible on the eggshell. However, this information is not verifiable.

## CONCLUSION

MS infection in chicken has noticeable economic significance in commercial poultry. Some of the MS strains are able to produce EAA leading to a significant decrease in eggshell strength, egg breakage, and general decrease in egg production. In poultry industry it is very important to detect egg quality problem earlier.

The method described in the article can support the detection of egg lesions in MS positive flocks. It is enough to collect waste in the form of eggshells, perform spectral measurements, and apply the classification procedure presented in the article.

Initial research on 120 eggshells (2,541 measurements) was carried out. Half of eggs came from MS-infected chickens. Graphs of spectral transmittance from areas of about 1 mm^2^ each were analyzed. Based on the analysis of transmittance spectra, a series of parameters describing the geometric features of the spectrum was created. They were the basis to create three decision trees (one for mixed colors of eggshells, one for brown eggshells and one for white), implemented to pre-determine whether a tested egg belongs to a group of chicken with an increased risk of being MS-infected. The presented classification method, after applying 5-fold cross-validation, was characterized by accuracy at the level of 84, 88, and 84%, respectively. The best results were obtained for the classifier based on brown shells. The smallest FNR = 13% was also achieved in this group. Minimizing FNR and maximizing specificity and accuracy is difficult due to the possibility of an incorrect determination of a shell before the spectral measurement (not all the eggs from a farm considered infected must come from sick hens).

In order to make a final decision on the possibility of commercialization of the presented method for distinguishing the uninfected chickens eggs from the MS-infected chickens eggs, it is necessary to perform tests on a larger number of samples.
